# Supplementary material for: Development of a Specific Nested PCR Assay for the Detection of 16SrI Group Phytoplasmas Associated with Sisal Purple Leafroll Disease in Sisal Plants and Mealybugs
Source: Plants (Basel). 2022 Oct 23;11(21):2817. doi: 10.3390/plants11212817 (PMC9658197; doi:10.3390/plants11212817)
Supplement: Supplementary file 1 [file plants-11-02817-s001.zip › Figure S1.pdf]

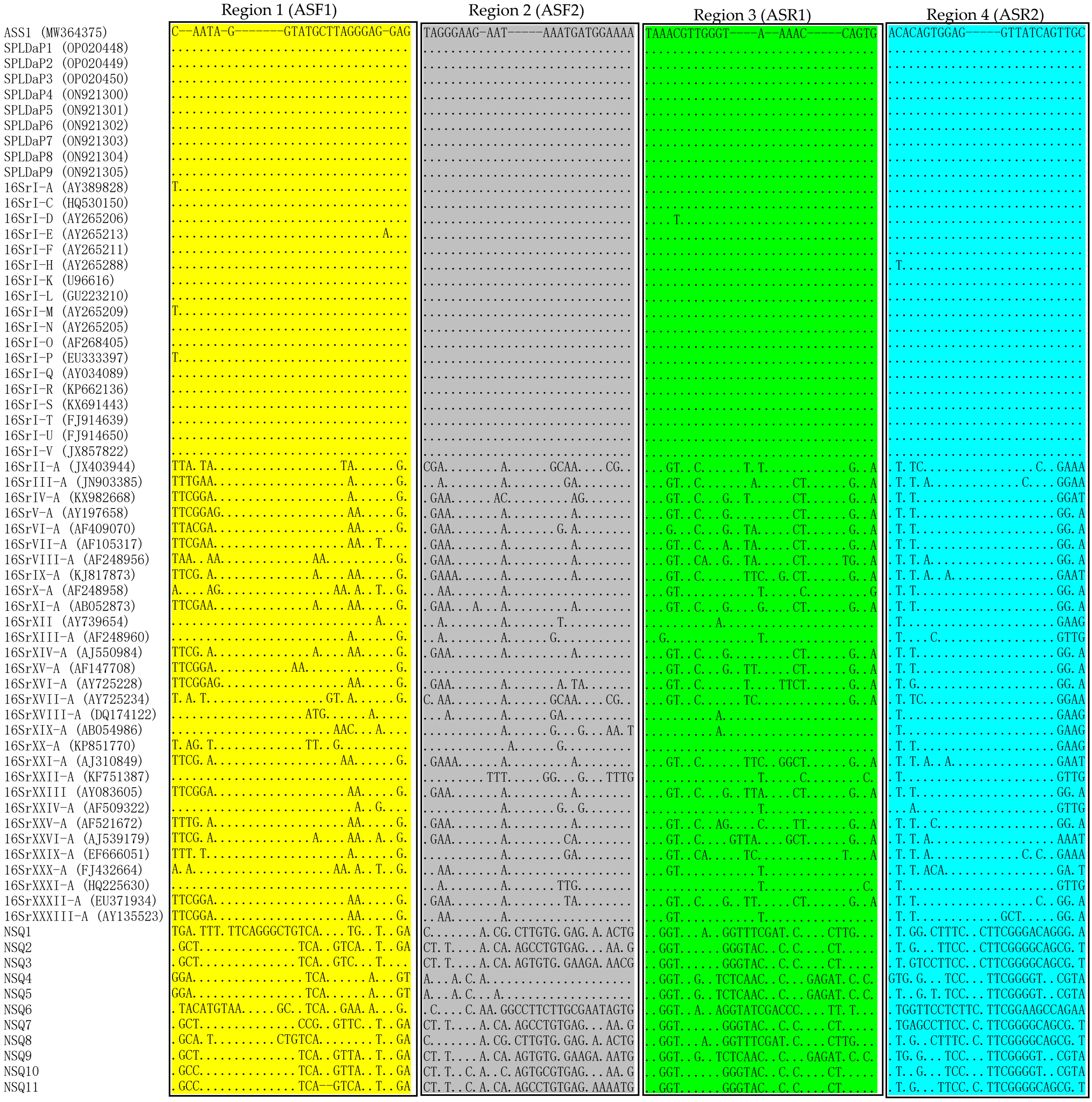

Figure S1. Alignment of 16S rRNA gene nucleotide sequences from 10 SPLDaP (16SrI-B) (ASS1, SPLDaP1 to SPLDaP9), 18 subgroups of 16SrI (-A,-C to -V), 30 representative 16Sr groups (16SrII to 16SrXXXIII), as well as 11 non-target bacterial species (NSQ1- NSQ11) amplified from the sisal in the 5'-to-3' direction, and four regions (1-4) of sequence polymorphism were trimmed. The primers ASR1 and ASR2 is the reverse compliment to the region (3, 4) highlighted in Figure S1. '----' represented spacer sequence, '....' represented same sequence as target primer.
