# Supplementary material for: Development of a Specific Nested PCR Assay for the Detection of 16SrI Group Phytoplasmas Associated with Sisal Purple Leafroll Disease in Sisal Plants and Mealybugs
Source: Plants (Basel). 2022 Oct 23;11(21):2817. doi: 10.3390/plants11212817 (PMC9658197; doi:10.3390/plants11212817)
Supplement: Supplementary file 1 [file plants-11-02817-s001.zip › Figure S2.pdf]

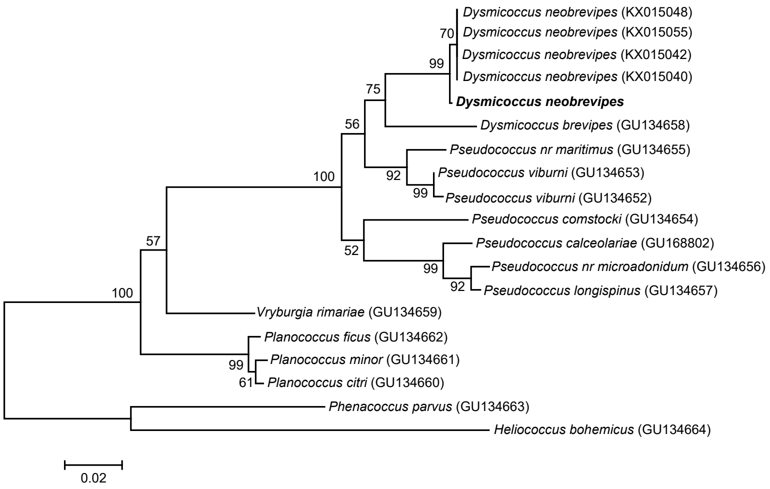

Figure S2. Phylogenetic analysis of mealybugs collected from sisal in South China. The 28S gene sequence of the mealybugs was amplified by PCR, and a phylogenetic tree was constructed using MEGA 6. The phylogenetic tree constructed by the neighbor-joining method. Numbers on branches are the bootstrap values (1000 replicates). The bold represented the 28S gene sequence amplified from *Dysmicoccus neobrevipes* in this study.
