# Supplementary material for: Development of a Specific Nested PCR Assay for the Detection of 16SrI Group Phytoplasmas Associated with Sisal Purple Leafroll Disease in Sisal Plants and Mealybugs
Source: Plants (Basel). 2022 Oct 23;11(21):2817. doi: 10.3390/plants11212817 (PMC9658197; doi:10.3390/plants11212817)
Supplement: Supplementary file 1 [file plants-11-02817-s001.zip › Figure S3.pdf]

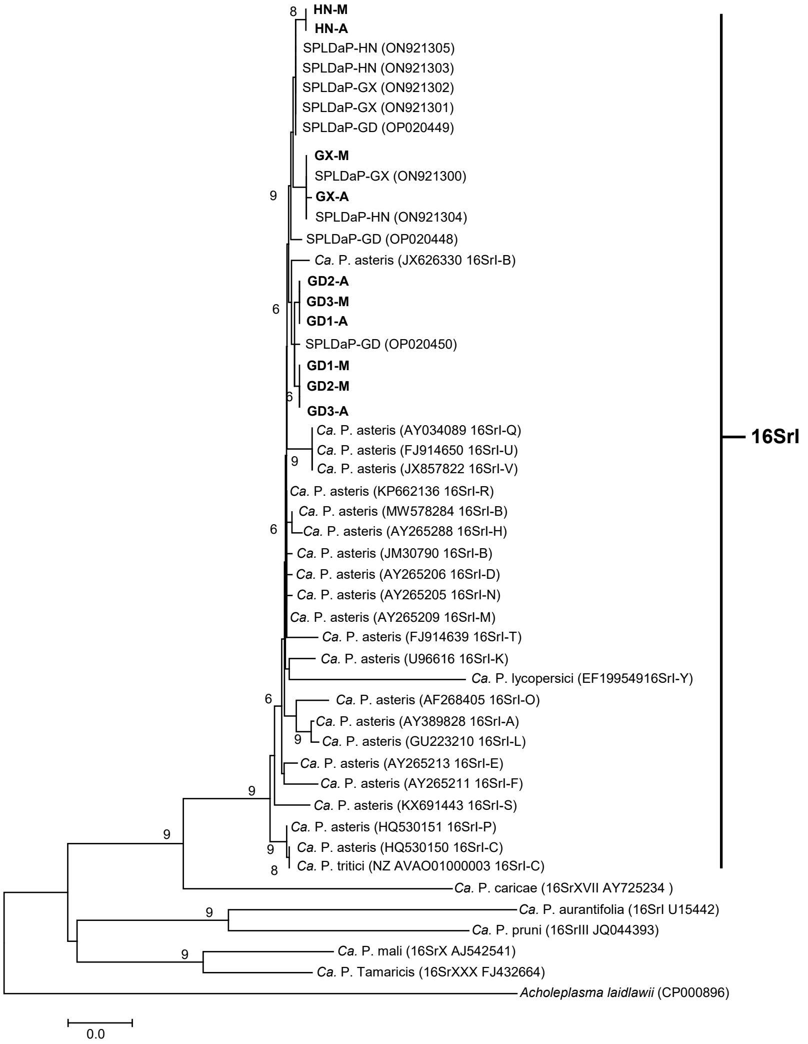

Figure S3. A phylogenetic tree constructed of 16S rRNA gene sequences from phytoplasma by the neighbor-joining method using MEGA 6. Numbers on branches are the bootstrap values (1000 replicates). GenBank accession numbers are given in parentheses, and *Acholeplasma Laidlawii* was the outgroup. The bold represented the F2nR2 regions sequences of 16Sr RNA gene amplified from SPLDaP in SPLD leaf samples (-A) and corresponding mealybugs samples (-M) from Hainan (HN), Guangdong (GD) and Guangxi (GX).
