# Supplementary material for: Development of a Specific Nested PCR Assay for the Detection of 16SrI Group Phytoplasmas Associated with Sisal Purple Leafroll Disease in Sisal Plants and Mealybugs
Source: Plants (Basel). 2022 Oct 23;11(21):2817. doi: 10.3390/plants11212817 (PMC9658197; doi:10.3390/plants11212817)
Supplement: Supplementary file 1 [file plants-11-02817-s001.zip › Figure S4.pdf]

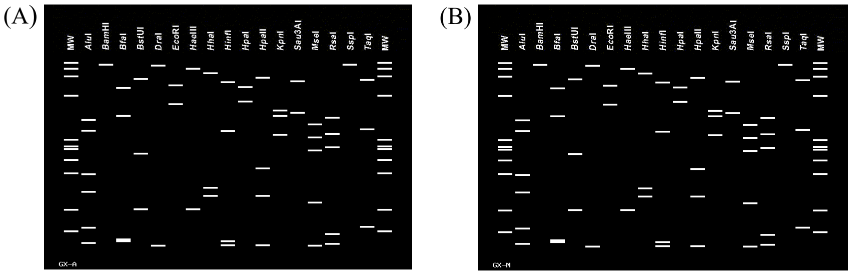

Figure S4. Virtual RFLP patterns generated from fragments of 16S rRNA genes of SPLDaP amplified from sisal plants (A) and mealybugs (B). Recognition sites for the following 17 restriction enzymes were used in the simulated digestions: *Alu*I, *Bam*HI, *Bfa*I, *Bst*UI (*Tha*I), *Dra*I, *Eco*RI, *Hae*III, *Hha*I, *Hinf*I, *Hpa*I, *Hpa*II, *Kpn*I, *Sau*3AI (*Mbo*I), *Mse*I, *Rsa*I, *Ssp*I and *Taq*I.
