# Supplementary material for: Development of a Specific Nested PCR Assay for the Detection of 16SrI Group Phytoplasmas Associated with Sisal Purple Leafroll Disease in Sisal Plants and Mealybugs
Source: Plants (Basel). 2022 Oct 23;11(21):2817. doi: 10.3390/plants11212817 (PMC9658197; doi:10.3390/plants11212817)
Supplement: Supplementary file 1 [file plants-11-02817-s001.zip › Table S1.pdf]

Table S1. 28S gene sequence identities of mealybugs in this study and other mealybug species.

**Species**	**GenBank Accession nos.**	**Sequences identity（%）to mealybugs in this study**
Dysmicoccus neobrevipes	KX015048	99.68%
Dysmicoccus neobrevipes	KX015042	99.68%
Dysmicoccus neobrevipes	KX015055	99.68%
Dysmicoccus neobrevipes	KX015040	99.68%
Planococcus citri	GU134660	99.68%
Dysmicoccus brevipes	GU134658	94.43%
Pseudococcus viburni	GU134653	94.39%
Pseudococcus viburni	GU134652	94.08%
Pseudococcus nr maritimus	GU134655	93.17%
Pseudococcus calceolariae	GU168802	90.31%
Pseudococcus longispinus	GU134657	90.06%
Pseudococcus nr microadonidum	GU134656	90.06%
Pseudococcus comstocki	GU134654	89.81%
Vryburgia rimariae	GU134659	86.05%
Planococcus minor	GU134661	85.14%
Planococcus ficus	GU134662	84.88%
Phenacoccus parvus	GU134663	77.84%
