# Supplementary material for: Development of a Specific Nested PCR Assay for the Detection of 16SrI Group Phytoplasmas Associated with Sisal Purple Leafroll Disease in Sisal Plants and Mealybugs
Source: Plants (Basel). 2022 Oct 23;11(21):2817. doi: 10.3390/plants11212817 (PMC9658197; doi:10.3390/plants11212817)
Supplement: Supplementary file 1 [file plants-11-02817-s001.zip › Table S2.pdf]

Table S2. The numbers of sisal samples collected in south China between 2018 and 2021.

Location	Collected Area	Number of samples	
		**Symptomatic samples**	**Asymptomatic samples**
**Hainan Province**			
Haikou City	Epidemic	15	10
Changjiang Country	Epidemic	5	5
**Guangdong Province**			
Xuwen Country	Epidemic	20	20
Leizhou Country	Epidemic	10	10
Zhanjiang City	Non-epidemic	-[1]	10
**Guangxi Province**			
Qinzhou City	Epidemic	30	20
Chongzuo City	Non-epidemic	-	10
Nanning City	Non-epidemic	-	10

[1] No samples were collected.
